# Mortality, Recurrence, and Dependency Rates Are Higher after Acute Ischemic Stroke in Elderly Patients with Diabetes Compared to Younger Patients

**DOI:** 10.3389/fnagi.2016.00142

**Published:** 2016-06-16

**Authors:** Xue Long, Yongzhong Lou, Hongfei Gu, Xiaofei Guo, Tao Wang, Yanxia Zhu, Wenjuan Zhao, Xianjia Ning, Bin Li, Jinghua Wang, Zhongping An

**Affiliations:** ^1^Department of Neurology, Tianjin Haibin People’s HospitalTianjin, China; ^2^The Graduate School, Tianjin University of Traditional Chinese MedicineTianjin, China; ^3^Department of Neurology, Tianjin Huanhu HospitalTianjin, China; ^4^Department of Neurology, Tianjin Medical University General HospitalTianjin, China; ^5^Department of Epidemiology, Tianjin Neurological InstituteTianjin, China

**Keywords:** ischemic stroke, diabetes, outcomes, elderly, mortality, dependency, recurrence

## Abstract

Stroke has a greater effect on the elderly than on younger patients. However, the long-term outcomes associated with stroke among elderly patients with diabetes are unknown. We aimed to assess the differences in long-term outcomes between young and elderly stroke patients with diabetes. A total of 3,615 acute ischemic stroke patients with diabetes were recruited for this study between 2006 and 2014. Outcomes at 12 and 36 months after stroke (including mortality, recurrence, and dependency) were compared between younger (age <75 years) and elderly (age ≥75 years) patients. The elderly group included 692 patients (19.1%) overall. Elderly patients were more likely than younger patients to have a Trial of Org 10172 in Acute Stroke Treatment classification of stroke due to cardioembolism, moderate and severe stroke, and atrial fibrillation, but less likely to have hypertension and dyslipidemia, current smokers, and alcohol consumers. Mortality, dependency, and recurrence rates at 12 months after stroke were 19.0, 48.5, and 20.9% in the elderly group and 7.4, 30.9, and 15.4% in the younger group, respectively (all *P* < 0.05). Corresponding rates at 36 months after stroke were 35.4, 78.7, and 53.8% in the elderly group and 13.7, 61.7, and 43.0% in the younger group, respectively (all *P* < 0.001). The mortality, dependency, and recurrence rates at 12 and 36 months after stroke were significantly higher in the elderly group than in the younger group after adjusting for stroke subtypes, stroke severity, and risk factors. Odds ratios (95% confidence interval) at 12 and 36 months after stroke were 2.18 (1.64–2.89) and 3.10 (2.35–4.08), respectively, for mortality, all *P* < 0.001; 1.81 (1.49–2.20) and 2.04 (1.57–2.34), respectively, for dependency, all *P* < 0.001; and 1.37 (1.06–1.76) and 1.40 (1.07–1.85), respectively, for recurrence, *P* = 0.016. The findings from this study suggest that management and secondary prevention should be emphasized in elderly patients with diabetes in China to reduce mortality, recurrence, and dependency after stroke.

## Introduction

Age is the most important non-modifiable risk factor for all subtypes of stroke, particularly ischemic stroke (MONICA Project (Monitoring Trends Determinants in Cardiovascular Disease), 1988; Feigin et al., 2014). The incidence of stroke more than doubles in each successive decade after the age of 55 years (MONICA Project (Monitoring Trends Determinants in Cardiovascular Disease), 1988). The age-standardized stroke incidence in low- and middle-income countries exceeds that in high-income countries by 21% in individuals ≥75 years old, and age-standardized stroke mortality exceeds that in high-income countries by 33%. In addition, the number of lost disability-adjusted life-years is 1.5 times higher in low- and middle-income countries (Rothwell et al., 2005). China has experienced significant growth in its elderly population in recent decades; those ≥75 years old accounted for 3.5% of the population in 2013, and there were 200 million elderly residents ≥65 years old in 2014 (National Bureaus of Statistics of the People’s Republic of China, 2014; Peilin et al., 2014).

Population-based studies have indicated that 65% of all strokes occur in individuals >65 years old (MONICA Project (MONICA Project (Monitoring Trends Determinants in Cardiovascular Disease), 1988; Lloyd-Jones et al., 2009). Moreover, elderly patients have worse functional outcomes after stroke than do younger patients, and these differences persist even after adjusting for stroke risk factors and other comorbidities (Di Carlo et al., 1999; Kelly-Hayes et al., 2003; Bhalla et al., 2004; Kammersgaard et al., 2004; Palnum et al., 2008). A population-based study demonstrated that the incidence of first-ever stroke increased by 4.3% annually in China among those ≥65 years old from 1992 to 2012 (Wang et al., 2015).

It is well known that diabetes is significantly associated with stroke, and patients with diabetes mellitus (DM) are at greater risk of stroke than individuals without diabetes (Brott et al., 1989; Recommendations on Stroke Prevention, Diagnosis, and Therapy, 1989; Ullberg et al., 2015). A few studies have investigated the association between DM and long-term prognosis of patients following acute ischemic stroke (AIS; Rankin, 1957; Mahoney and Barthel, 1965); however, the evidence for this association among elderly patients remains controversial. A report from China demonstrated that DM independently predicted poor outcomes within 6 months after AIS in Chinese patients (Wang et al., 2013). However, differences in long-term outcomes among elderly stroke patients with DM have not been established.

Therefore, in this study, we aimed to evaluate differences in long-term outcomes between younger patients (<75 years old) and elderly patients (≥75 years old) with diabetes following AIS from a hospital-based stroke registry in Tianjin, China.

## Materials and Methods

All consecutive patients with AIS who were hospitalized in three stroke units in Tianjin, China between January 2006 and September 2014 were recruited for this study. Stroke was defined according to the World Health Organization’s criteria, and a diagnosis of AIS was confirmed in all patients based on neuroimaging evidence (Banks and Marotta, 2007). Those patients diagnosed with transient ischemic attack were excluded. DM was defined by a previous or new-onset physician diagnosis of DM, or use of hypoglycemic medications.

The ethics committee of Tianjin Medical University General Hospital approved the study, and patients or their next of kin provided written informed consent for participation in accordance with the Declaration of Helsinki.

Data were collected prospectively according to a standardized procedure. Ischemic stroke subtypes, neurological deficits, level of disability at the time of admission, stroke severity, and stroke risk factors were recorded. Levels of total cholesterol (TC), triglycerides (TG), high-density lipoprotein cholesterol (HDL-C), low-density lipoprotein cholesterol (LDL-C), fasting glucose (FG), and glycosylated hemoglobin (HbA1) at admission also were recorded. During the follow-up period, data on AIS outcomes, including all-cause death, dependency, and recurrence were assessed.

According to the Trial of Org 10172 in Acute Stroke Treatment (TOAST) criteria, ischemic stroke subtypes were classified according to etiology as stroke due to large artery atherothrombosis (LAA), stroke due to cardioembolism (CE), stroke due to small artery occlusion (SAO), stroke of other determined etiology, and stroke of undetermined etiology (Murray and Lopez, 1997). Neurological deficits were assessed using the National Institutes of Health stroke scale (NIHSS; Marini et al., 2004), and the level of disability was measured using the Barthel index (BI; Gur et al., 2012) and modified Rankin scale (mRS; Olindo et al., 2003). Stroke severity was categorized into three groups based on the NIHSS score: mild (NIHSS: ≤7), moderate (NIHSS: 8–16), and severe (NIHSS: ≥17) (Deng et al., 2012).

Risk factors for stroke included a medical history of hypertension, dyslipidemia, atrial fibrillation (AF), and obesity (body mass index ≥30 kg/m^2^); and modifiable lifestyle factors, including current smoking status and alcohol consumption.

Outcome measures for stroke were mortality, recurrence, and dependency rates 12 and 36 months after AIS. Death was defined as all-cause mortality at the corresponding time points after hospital admission. Stroke recurrence was defined as any new-onset vascular event, including stroke, myocardial infarction, and venous thrombosis, at least 30 days after stroke. Dependency was defined as an mRS score ≥3 (Fonarow et al., 2010). Mortality rates at 12 and 36 months after AIS were calculated as the number of all-cause deaths within 12 and 36 months divided by the total number of patients at the corresponding time points. The recurrence rate was calculated as the proportion of patients with new-onset vascular events, including patients who died of vascular events, during the follow-up period. The dependency rate was calculated as the number of patients with an mRS score ≥3 divided by the total number of stroke survivors at 12 and 36 months after AIS.

All patients were categorized into two groups by age: younger group (<75 years old) and elderly group (≥75 years old). All continuous variables, which included age, NIHSS, BI, mRS, and laboratory values (TC, TG, HDL-C, LDL-C, FG, and HbA1) are presented as the mean (standard deviation) or median (interquartile range, IQR) and compared between groups using the Student’s *t*-test or Mann–Whitney *U* test. All categorical variables, including ischemic stroke subtype, stroke severity, and stroke risk factors, are presented as the number (percentage); the differences between groups for categorical variables were analyzed using the Chi-squared test. A multivariate logistic regression analysis was performed to assess factors associated with stroke outcomes by adjusting for covariates (including stroke subtype, severity, and risk factors). The results of the multivariate analysis are presented as adjusted odds ratios (ORs) with 95% confidence intervals (CIs). All statistical analyses were performed using SPSS version 15.0 (SPSS Inc., Chicago, IL, USA), and *P*-values <0.05 resulting from two-tailed tests were considered statistically significant.

## Results

**FIGURE 1:**
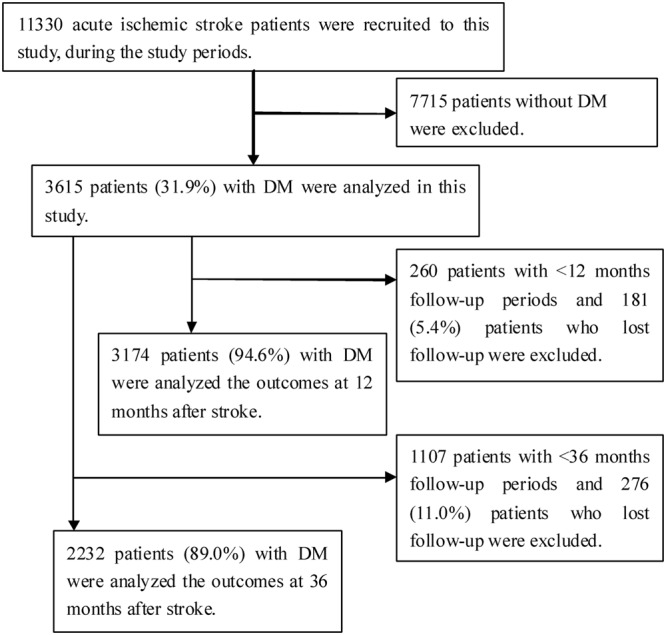
**Flow diagram of patients’ selection**.

During the study period, 11,330 consecutive patients with AIS were hospitalized in three stroke units. Of these patients, 7,715 patients (68.1%) were excluded because they did not have DM; therefore, 3,615 patients (31.9%) were included in the study. A total of 3,174 patients (94.6%) were analyzed at 12 months after excluding 181 patients (5.4%) who were lost to follow-up among 3,355 patients with a sufficient follow-up time of 12 months. Similarly, 2,232 patients (89.0%) were analyzed at 36 months after excluding 276 patients (11.0%) who were lost to follow-up among 2,508 patients with a sufficient follow-up time of 36 months (**Figure 1**).

The elderly group included 692 patients (19.1%), more than half of whom (53.6%) were men. Elderly patients were more likely than younger patients to have a TOAST classification of CE (9.6 vs. 2.5%; *P* < 0.00001). The proportion of patients with moderate and severe stroke were significantly higher in the elderly group than in the younger group (31.5 vs. 26.4%, *P* = 0.007 for moderate stroke; 13.7 vs 8.0%, *P* < 0.00001 for severe stroke). Similarly, median NIHSS and mRS scores were higher, whereas BI scores were lower, in elderly patients compared with younger patients (all *P* < 0.001, **Table 1**).

**Table 1:** Demographical and clinical characteristics in ischemic stroke patients with DM by age.

Characteristics	Younger group	Elderly group	*P*
Gender, *n* (%):	2,923 (80.9)	692 (19.1)	<0.001
Men	1,852 (83.3)	371 (16.7)	
Women	1,071 (76.9)	321 (23.1)	
Total			
TOAST classification, *n* (%):			
Atherothrombotic	2,066 (71.1)	467 (68.9)	0.099
Small artery disease	678 (23.3)	139 (20.5)	0.079
Cardiac embolism	73 (2.5)	65 (9.6)	<0.0001
Others determined etiology	47 (1.6)	2 (0.3)	0.007
Undetermined etiology	42 (1.4)	5 (0.7)	0.136
Stroke severity, *n* (%):			
Mild	1,918 (65.6)	379 (54.8)	<0.0001
Moderate	772 (26.4)	218 (31.5)	0.007
Severe	233 (8.0)	95 (13.7)	<0.0001
Neurological function:			
NIHSS	6 (6)	7 (9)	<0.001
BI	60 (50)	50 (50)	<0.001
mRS	3 (2)	4 (1)	<0.001
Laboratory examination:			
Total cholesterol	5.04 (1.13)	5.01 (1.20)	0.533
Triglyceride	1.93 (1.44)	1.56 (0.84)	<0.001
High-density lipoprotein cholesterol	1.01 (0.25)	1.05 (0.26)	<0.001
Low-density lipoprotein cholesterol	3.10 (0.88)	3.11 (0.94)	0.668

The prevalence of hypertension, dyslipidemia, current smoking, and alcohol consumption was lower in elderly patients compared to younger patients (**Table 2**); however, an opposite trend was observed for the prevalence of AF (13.6 vs. 4.9%, *P* < 0.001). There were no significant differences in the prevalence of artery stenosis and obesity between the elderly and younger patients.

**Table 2:** The prevalence of risk factors in ischemic stroke patients with DM by age.

Previous history of diseases and risk factors	Younger group	Elderly group	*P*
Hypertension	2,298 (78.6)	515 (74.4)	0.017
Atrial fibrillation	144 (4.9)	94 (13.6)	<0.00001
Artery stenosis	804 (27.5)	186 (26.9)	0.739
Dyslipidemias	1,135 (38.8)	202 (29.2)	<0.001
Obesity	487 (16.7)	130 (18.8)	0.182
Current smoking	1,078 (36.9)	110 (15.9)	<0.001
Alcohol consumption	522 (17.9)	32 (4.6)	<0.001

Mortality, dependency, and recurrence rates at 12 months after stroke were 19.0, 48.5, and 20.9% in the elderly group and 7.4, 30.9, and 15.4% in the younger group, respectively (all *P* < 0.05). The corresponding rates at 36 months after stroke were 35.4, 78.7, and 53.8% in the elderly group and 13.7, 61.7, and 43.0% in the younger group, respectively (all *P* < 0.001). After adjusting for stroke subtypes, severity, and risk factors, mortality, dependency, and recurrence rates at 12 and 36 months after stroke were significantly higher in the elderly group than in the younger group. OR (95% CI) at 12 months after stroke was 2.18 (1.64–2.89, *P* < 0.001) for mortality, 1.81 (1.49–2.20, *P* < 0.001) for dependency, and 1.37 (1.06–1.76, *P* = 0.016) for recurrence, respectively. The corresponding values at 36 months after stroke were 3.10 (2.35–4.08, *P* < 0.001) for mortality, 2.04 (1.57–2.34, *P* < 0.001) for dependency, and 1.40 (1.07–1.85, *P* = 0.016) for recurrence, respectively (**Table 3**).

**Table 3:** The OR of mortality, recurrence, and dependency at 12 and 36 months after stroke among stroke patients with DM by age^∗^.

Outcomes	Younger group	Elderly group	Unadjusted		Adjusted^†^	
			OR (95% CI)	*P*	OR (95% CI)	*P*
12 months:						
Mortality	189 (7.4)	116 (19.0)	2.95 (2.30–3.79)	<0.001	2.18 (1.64–2.89)	<0.001
Dependency	786 (30.7)	296 (48.5)	2.13 (1.78–2.55)	<0.001	1.81 (1.49–2.20)	<0.001
Recurrence	367 (15.4)	102 (20.9)	1.45 (1.14–1.86)	0.003	1.37 (1.06–1.76)	0.016
36 months:						
Mortality	248 (13.7)	151 (35.4)	3.44 (2.70–4.36)	<0.001	3.10 (2.35–4.08)	<0.001
Dependency	1,113 (61.7)	336 (78.7)	2.30 (1.79–2.95)	<0.001	2.04 (1.57–2.64)	<0.001
Recurrence	636 (43.0)	141 (53.8)	1.54 (1.19–2.01)	<0.001	1.40 (1.07–1.85)	0.016

*^∗^OR indicated the risks in elderly group reference as younger group. ^†^Indicated adjusted by gender, stroke subtype, severity, and risk factors.*

## Discussion

In this study, we assessed and compared the outcomes and associated risk factors at 12 and 36 months after AIS between younger patients (<75 years old) and elderly patients (≥75 years old) with DM in China.

Diabetes mellitus is an independent risk factor for ischemic stroke, as the relative risk of ischemic stroke in patients with DM is 1.8–6.0 times higher than in patients without DM (Saposnik et al., 2008). Moreover, the prevalence of DM ranges from 21 to 44% in patients with AIS (Soares et al., 2011; Li et al., 2013). The China National Stroke Registry reported a 26.99% prevalence of DM overall (Schulz and Rothwell, 2003). Consistent with previous studies, we found a 31.9% prevalence of DM among patients with AIS, which was higher than the prevalence determined from the China National Stroke Registry. Different databases of stroke patients between studies may partly explain differences in the frequency of stroke patients with DM.

Previous studies have indicated that ischemic stroke greatly affects the older population (Lovett et al., 2004; Rothwell et al., 2005; Lloyd-Jones et al., 2009; Feigin et al., 2014). Several studies have reported different trends in the prevalence of hypertension, DM, and AF (Olindo et al., 2003; Bhalla et al., 2004; Marini et al., 2004; Gur et al., 2012) among elderly individuals. A study based on a multicenter stroke registry in China reported that patients aged 80 years and older were more likely to have a stroke with a TOAST classification of CE (11.6%). Furthermore, there were higher rates of hypertension (72.1%) and AF (23.4%) and lower rates of DM (17.5%), dyslipidemia (5.6%), current smoking status (22.8%), and heavy drinking (2.5%) among AIS patients ≥80 years old compared to patients <80 years old (Deng et al., 2012). Consistent with these studies, in this study, elderly patients exhibited different clinical features and stroke risk factors than younger patients. The proportions of patients with a TOAST classification of CE, severe stroke, and AF were higher in the elderly group than in the younger group. Younger patients were more likely to have a previous history of hypertension and dyslipidemia and were more likely to be current smokers and alcohol consumers than elderly patients. However, there was no significant difference with respect to artery stenosis or obesity between the two groups.

Previous studies have demonstrated that older patients have worse outcomes after stroke than do younger patients (Lovett et al., 2004; Kimura et al., 2005; Palnum et al., 2008; Tu et al., 2010, 2011). A study conducted in China reported a mortality rate of 34.78%, a dependency rate of 43.81%, and a recurrence rate of 36.02% at 12-month follow-up among AIS patients ≥80 years old (Deng et al., 2012). Another study reported that the 1-year case-fatality rate and disability rate in patients ≥80 years old were 33.8 and 37.8%, respectively (Tu et al., 2011). Findings from a multivariate analysis in another study revealed that TOAST classification of CE and LAA and moderate and severe stroke were associated with mortality and recurrence 3 months after stroke; moderate and severe stroke also were associated with dependency 3 months after stroke. In addition, moderate and severe stroke were significantly associated with mortality and dependency at 12 months (Bereczki et al., 2009).

Several studies have suggested that DM is associated with a higher stroke mortality rate (Zhang et al., 2008; Soares et al., 2011), but this association was not observed in other studies (Tuttolomondo et al., 2008; Weiss et al., 2013). Furthermore, many studies have indicated that DM was an important predictive factor for dependency (Zhang et al., 2008; Eriksson et al., 2012; Tanaka et al., 2013) and recurrence rates (Sun et al., 2009; Kong et al., 2010; Callahan et al., 2011) following stroke. However, studies indicated that treatment DM using antidiabetic before or after stroke onset has a meaningful effect on stroke outcome in patients with DM comparing those patients without DM (Kunte et al., 2007; Tziomalos et al., 2015). Age emerged as a highly significant inverse predictor of good functional outcome after ischemic stroke, it was independent of stroke severity, characteristics, and complications. (Knoflach et al., 2012). This study found that mortality, dependency, and recurrence rates were significantly higher in the elderly group than in the younger group among stroke patients with DM at both 12 and 36 months. The mortality, dependency, and recurrence rates in the elderly group were 2.2-, 1.8-, and 1.4-fold higher, respectively, than the corresponding rates in the younger group at 12 months, and were 3.1-, 2.0-, and 1.4-fold higher, respectively, than the corresponding rates in the younger group at 36 months. Age was a predictor of poor long-term outcomes among stroke patients with DM, it was independent of the benefit from treatment using antidiabetic. Worse long-term outcomes in elderly stroke patients with DM may be explained by the limited application of secondary measures for stroke prevention and poor health care services for elderly patients.

There are several limitations to this study. First, all patients were from the local hospital in Tianjin, China, resulting in a limited representation of the general population. Second, all patients were from the local university hospital or neurological specialty hospital, and accordingly, the proportion of patients with severe stroke was higher in this study than in previous studies. However, we adjusted the results by gender, stroke subtype, stroke severity, and risk factors in the multivariate analysis, thus addressing the effect of stroke severity on patient outcomes in our results. Third, pre-stroke information, including medication usage and complications such as pneumonia and urinary tract infection, were not analyzed in this study; therefore, we were unable to assess the impact of these factors on outcomes among elderly patients.

## Conclusion

This is the first study to assess the long-term (12 and 36 months) outcomes following AIS between younger and elderly AIS patients with DM in China. Mortality, dependency, and recurrence rates were significantly higher in the elderly group than in the younger group at both 12 and 36 months after AIS. The relationship between age and outcomes remained even after adjusting for stroke subtypes, severity, and risk factors. Age was a predictor of poor long-term outcomes among stroke patients with DM, it was independent of the benefit from treatment using antidiabetic. Poor outcomes in elderly patients may be partly explained by the higher frequency of women, less frequent use of secondary measures to prevent stroke, and poor health care within the elderly population. Thus, management and secondary prevention should be emphasized in elderly patients with DM in China to reduce mortality, recurrence, and dependency after stroke.

## Author Contributions

BL, JW, and ZA made substantial contributions to the conception and design of the study. XL, TW, YL, HG, XG, YZ, and WZ made substantial contributions to the acquisition, analysis, and interpretation of data. XN and JW substantially contributed to the analysis and interpretation of data. BL, JW, and ZA supervised the study. BL and ZA were involved in obtaining funding for the study. All authors contributed to the drafting of the manuscript and approved the final version.

## Conflict of Interest Statement

The authors declare that the research was conducted in the absence of any commercial or financial relationships that could be construed as a potential conflict of interest.
